# Chronic lead exposure reduces doublecortin-expressing immature neurons in young adult guinea pig cerebral cortex

**DOI:** 10.1186/1471-2202-13-82

**Published:** 2012-07-19

**Authors:** JuFang Huang, Kai Huang, Lei Shang, Hui Wang, Mengqi Zhang, Chun-Ling Fan, Dan Chen, Xiaoxin Yan, Kun Xiong

**Affiliations:** 1 Department of Anatomy and Neurobiology, Central South University School of Basic Medical Sciences, Changsha, Hunan, 410013, China; 2 Department of Anatomy, Shaoyang Medical College, Shaoyang, Hunan, 422000, China; 3 Grade 2006, Eight-year Medicine Doctor Program, Central South University Xiangya School of Medicine, Changsha, Hunan, 410013, China

**Keywords:** Immature neurons, Lead exposure, Doublecortin, Neocortex, Guinea pigs

## Abstract

**Background:**

Chronic lead (Pb) poisoning remains an environmental risk especially for the pediatric population, and it may affect brain development. Immature neurons expressing doublecortin (DCX+) exist around cortical layer II in various mammals, including adult guinea pigs and humans. Using young adult guinea pigs as an experimental model, the present study explored if chronic Pb exposure affects cortical DCX + immature neurons and those around the subventricular and subgranular zones (SVZ, SGZ).

**Results:**

Two month-old guinea pigs were treated with 0.2% lead acetate in drinking water for 2, 4 and 6 months. Blood Pb levels in these animals reached 10.27 ± 0.62, 16.25 ± 0.78 and 19.03 ± 0.86 μg/dL at the above time points, respectively, relative to ~3 μg/dL in vehicle controls. The density of DCX + neurons was significantly reduced around cortical layer II, SVZ and SGZ in Pb-treated animals surviving 4 and 6 months relative to controls. Bromodeoxyuridine (BrdU) pulse-chasing studies failed to find cellular colocalization of this DNA synthesis indicator in DCX + cells around layer II in Pb-treated and control animals. These cortical immature neurons were not found to coexist with active caspase-3 or Fluoro-Jade C labeling.

**Conclusion:**

Chronic Pb exposure can lead to significant reduction in the number of the immature neurons around cortical layer II and in the conventional neurogenic sites in young adult guinea pigs. No direct evidence could be identified to link the reduced cortical DCX expression with alteration in local neurogenesis or neuronal death.

## Background

As one of the ubiquitously polluted heavy metals in ecosystem and modern industry, lead (Pb) may enter human body via many routes including airway, water and fo od [1,2]. Pb exposure many cause chronic central and peripheral nerve damages in humans at all ages, with more concerns for the pediatric population. Neurological consequences of Pb poison in children are reported to include reduction in IQ and learning/memory capability, hearing and language impairments and certain neuropsychological abnormalities [3-11]. The cellular mechanism underlying Pb overexposure-induced neurotoxicity in the central nervous system is complex. Of interest, recent studies have shown that chronic low level Pb exposure may inhibit neurogenesis in the hippocampal formation and affect the differentiation/maturation of the newly-generated granule cells, which may be relevant to behavioral and cognitive impairments associated with Pb poisoning [12-14].

We and others have identified a novel group of immature neurons located around layer II of the adult cerebral cortex in relatively large mammals including guinea pigs, cats, rabbits, nonhuman primates and humans; these cells express doublecortin (DCX+) and other typical immature neuronal markers [15-24]. These cortical immature neurons are more numerous in young relative to more mature animals and appear to be involved in neuronal structural plasticity [17-19,25]. More recently, these immature neurons are documented in brains of human infants and children [21,25,26]. The present study attempted to determine whether these immature cortical neurons were vulnerable to chronic Pb exposure using young adult guinea pigs as an experimental model. Data were also compared to DCX + neurons in the subventricular zone (SVZ) and subgranular zone (SGZ) that relate to adult neurogenesis [24]. Efforts were also taken to explore if changes in cortical DCX expression overtly relate to local neurogenesis and neuronal death.

## Results

### Elevation of blood Pb levels following chronic lead exposure

**Figure 1:**
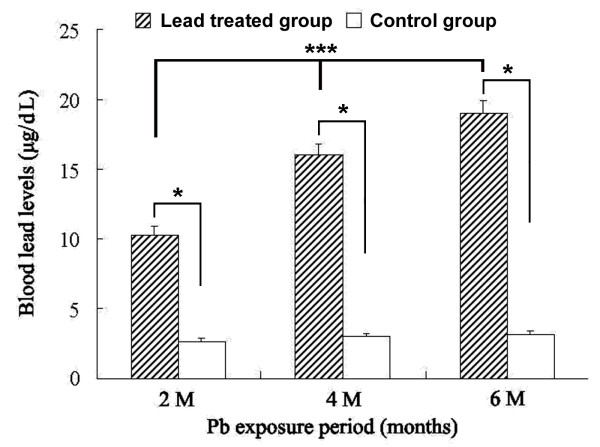
**Elevation of blood Pb levels in adult guinea pigs subjected to Pb exposure for 2, 4 and 6 months.** Each bar represents the mean ± SEM. ***: *p*<0.01 among different Pb exposure time groups, F = 103.352, one-way ANOVA. *: *p*<0.05 between Pb exposure group *vs* same age-matched control group (post hoc tests).

Blood Pb levels were increased to 10.27 ± 0.62 μg/dL in the 2 month Pb exposure group, to 16.25 ± 0.78 μg/dL in the 4 month surviving group, and to 19.03 ± 0.86 μg/dL in the 6 month surviving group. Among the age-matched control groups, blood Pb levels were 2.65 ± 0.21 μg/dL, 3.01 ± 0.25 μg/dL, 3.13 ± 0.31 μg/dL, respectively. Statistically, blood Pb levels elevated in the animal groups treated with lead acetate in a time-dependent manner at the 3 time points relative to age-matched control groups, with posthoc tests indicating no differences among the control groups (Figure 1).

### Decline in DCX + cells in cortical layer II following chronic lead exposure

**Figure 2:**
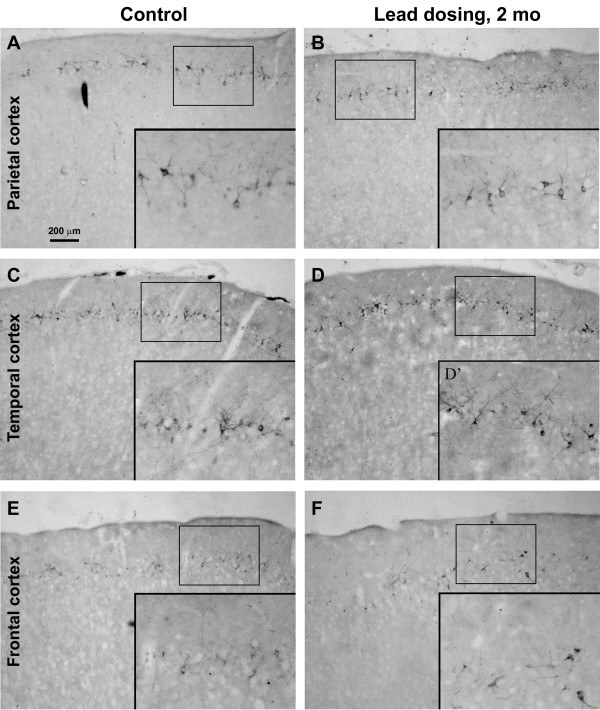
**Doublecortin-immunoreactive (DCX+) cells in different cortical areas from Pb exposure and age-matched control groups surviving 2 months.** Left panel images show parietal (**A**), temporal (**C**) and frontal (**E**) cortices from a control animal. Right panel images (**B**, **D**, **F**) show images from comparable regions in a Pb-treated animal. Inserts show closer view of the labeled cells, with arrows pointing to medium to large sized, and small sized cells. Overall, the amount and morphology of DCX + cells appear to be comparable between the two groups. Scale bar in panel A = 200 μm applying to main panels, equivalent to 100 μm for enlarged inserts.

**Figure 3:**
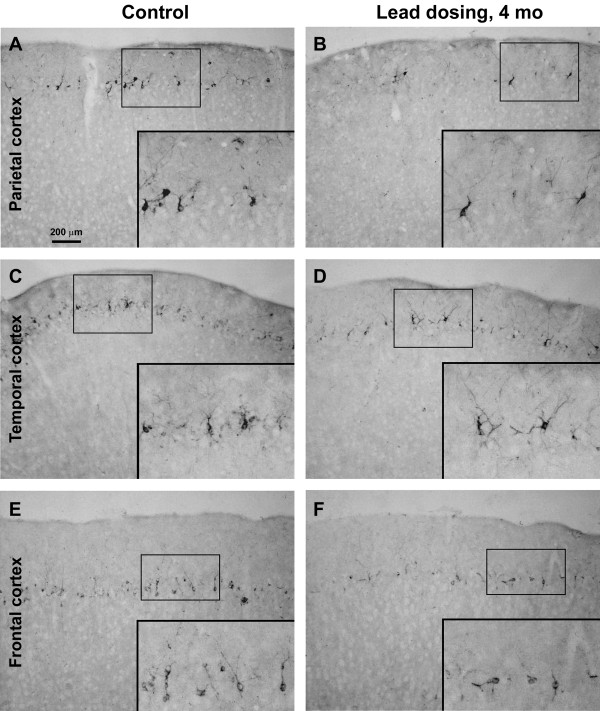
**Doublecortin-immunoreactive (DCX+) cells in representative cortical areas from a Pb-treated (right panels) and an age-matched control (left panels) guinea pigs surviving 4 months.** The amount of DCX + cells are noticeably reduced in the parietal (**A**, **B**), temporal (**C**, **D**) and frontal (**E**, **F**) cortices in the Pb treated animal relative to control. Inserts are enlarged to illustrate DCX + cells with varying sizes and morphologies. Scale bar in (**A**) = 200 μm applying to main image panels, equivalent to 100 μm for inserts.

**Figure 4:**
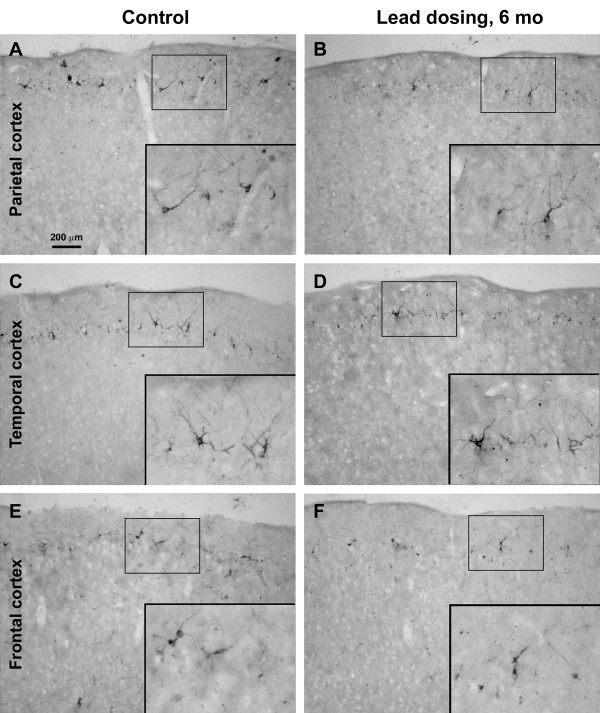
**Doublecortin-immunoreactive (DCX+) cells in different cortical areas from Pb exposure (right panels) and age-matched control (left panels) groups surviving 6 months.** A dramatic loss of DCX + cells occur in the Pb-treated relative to control animals over that parietal (**A**, **B**), temporal (**C**, **D**) and frontal (**E**, **F**) cortex. Scale bar in panel **A** = 200 μm applying to main panels, equivalent to 100 μm for enlarged inserts.

DCX + cells were found in multiple cortical areas of the guinea pigs, including Pb exposure and age-matched control groups, as with previous reports [17,20]. Thus, distinctly labeled DCX + cells with varying sizes and morphology formed a cellular band deep to layer I over the frontal, parietal, temporal and occipital cortical areas. By visual comparison DCX + cells in Pb exposure groups appeared to be reduced over all cortical areas in animals survived 4 and 6 months, but not in those survived for 2 months, relative to age-matched control groups (Figures 2,3 and 4).

**Figure 5:**
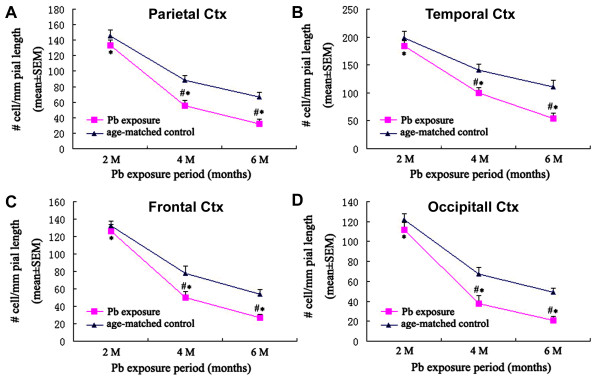
**Densitometric data showing a time-related decline of doublecortin-immunoreactive (DCX+) cells, expressed as number of cells per 1 mm pial distance, in different cortical areas from Pb exposure relative to age-matched control group guinea pigs.A**: parietal cortex; **B**: temporal cortex; **C**: frontal cortex; **D**: occipital cortex. Each bar represents the mean ± SEM. #: *p*<0.05 for Pb exposure *vs* age-matched control groups; *: *p*<0.05 among different Pb exposure time groups, F^a^ = 99.269, F^b^ = 100.500, F^c^ = 219.743, F^d^ = 105.450.

**Figure 6:**
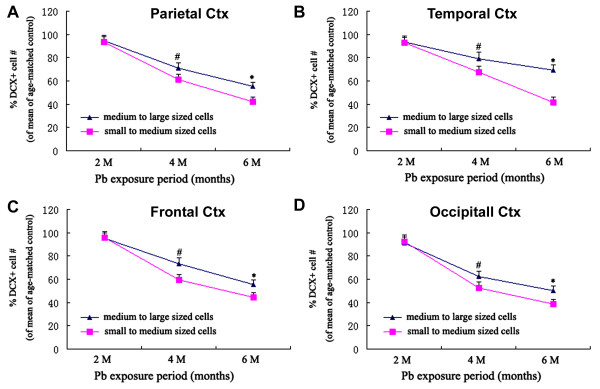
**Line graphs showing the extent of reduction (% of control levels) of smaller (blue) relative to larger (purple) doublecortin-immunoreactive (DCX+) cells in Pb-treated guinea pigs over the parietal (A), temporal (B), frontal (C) and occipital (D) cortices surviving 2, 4 and 6 months.** Statistic differences exist between of the two cell groups at 4 and 6 months surviving points in all analyzed areas. #: *p*<0.05; *: *p*<0.01.

The densities of DCX immunoreactive cells were quantified in the frontal, parietal, temporal and occipital areas. Cell counting in the frontal cortex was performed on 3 equally-spaced (720 μm apart) coronal sections passing the striatum, with the first one at the level of the first appearance of the anterior horn. Cell counting in the parietal and temporal cortices were conducted in 3 equally-spaced (also 720 μm apart) coronal sections around the “temporal pole” or the widest portion of the cerebrum, using the piriform fissure and lateral sulcus as landmarks for dividing the temporal and parietal areas [17]. Cell counting in the occipital cortex was carried out in 3 equally-spaced (720 μm apart) coronal sections passing the superior colliculus. Compared to age-matched control groups, the density of the entire DCX + cell population around layer II was significantly declined in the lead-treated groups surviving 4 and 6 months surviving groups over the parietal (Figure 5A), temporal (Figure 5B), frontal (Figure 5C) and occipital (Figure 5D) cortices. However, no significant reductions were detected in these areas at 2 months following Pb dosing relative to control (Figure 5A-D). The densitometric analyses on DCX + cells based on somal sizes indicated that the smaller (<10 μm in longer somal diameter) DCX + cells were decreased more significantly relative to larger ones (>10 μm in longer somal diameter) in all of the 4 analyzed areas (Figure 6).

### Decline in DCX + cells in the SVZ and SGZ following chronic lead exposure

**Figure 7:**
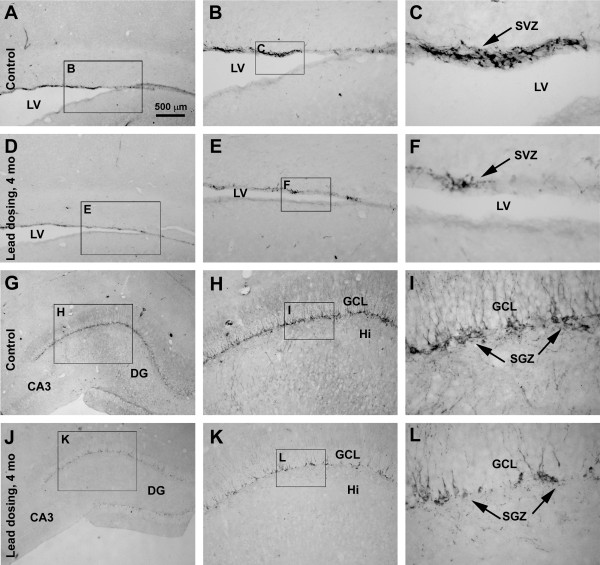
**Representative data showing doublecortin-immunoreactive (DCX+) cells in the subventricular zone (SVZ) (A-F) and subgranular zone (SGZ) (G-L) in age-matched control (A-C; G-I) compared to Pb-treated (D-F; J-L) animals surviving 4 months.** Framed areas in left panel images are sequentially enlarged as middle and right panel images. Scale bar in (A) = 200 μm applying to left panels; equal to 200 μm for middle panels and 50 μm for right panels.

Consistent with previous reports regarding lead-induced decrease in adult neurogenesis in small laboratory rodents [12-14], the present study also noted a significant decline of DCX + neurons in the subventricular zone (SVZ) and subgranular zone (SGZ) in animals subjected to chronic lead exposure relative to controls (Figure 7A-F). Thus, there was a dramatic loss of DCX + cells around the SVZ in the Pb-treated animals surviving 2, 4 and 6 months relative to controls. The same situation existed in the dentate gyrus wherein DCX + cells at the SGZ were apparently reduced in the Pb-treated relative to control guinea pigs (Figure 7G-L). Because DCX + cells were densely packed in the SVZ and SGZ, an analysis was carried by simply using the optic density of labeling along the SVZ and SGZ. The intensity of DCX immunoreactivity was reduced up to 50% (2 months) to 80% (4 and 6 months) in these two areas in the Pb-treated relative to controls (with *P* < 0.01 between the experimental and control groups at all time points) (quantification not shown).

### Lack of colocalization of BrdU in cortical DCX + neurons

**Figure 8:**
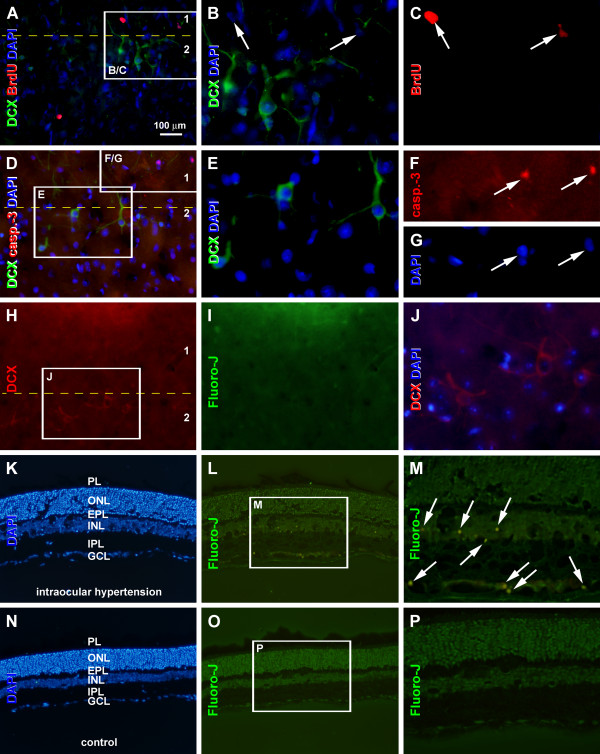
**Representative data showing that doublecortin-immunoreactive (DCX+) cells around neocortical layer II in Pb-treated animals surviving 6 months do not colocalize with BrdU (A-C), active caspase-3 (D-G) or Fluoro-Jade C labeling (H-J).** Markers and fluorescent channels are indicated on the left of each panel. Note that BrdU labeled cells (arrows) are present in layer I and upper III (**A**, **C**). Nuclei labeled for active caspase-3 are occasionally seen in layer I (**D**, **F**, and **G**). Fluoro-Jade C labeled cells are rarely detected in the cortex (**H**-**J**) or normal rat retina (**N**-**P**). In contrast, they are found in the rat retina 24 h after intraocular hypotension (**K**-**M**). PL: photoreceptor layer; ONL: outer nuclear layer; EPL: external plexiform layer; INL: inner nuclear layer; IPL: inner plexiform layer; GCL: ganglion cell layer. Scale bar = 100 μm in A applying to **D**, **H**, **I**, **K**, **L**, **N** and **O**; equal to 50 μm for **B**, **C**, **E**-**G**, **J**, **M** and **P**.

Recently studies have shown that the DCX + immature neurons around layer II could only occasionally be identified to colocalize with BrdU after pulse-chase administration of this DNA synthesis marker [27-32]. In the present study, double immunofluorescence was performed 2 and 4 months after BrdU injection. No clear BrdU colocalization with DCX or NeuN (data not shown) was found in either the Pb-treated guinea pigs or control animals in the 4 and 6 months surviving groups (Figure 8A-C). At both time points, singly labeled BrdU-containing cells were present in the superficial cortex, mostly over layers I and II (Figure 8A-C).

### Lack of active caspase-3 and fluoro-jade C labeling in cortical DCX + neurons

Active caspase-3 and Fluoro-Jade C labelings are used to detect apoptosis and cell injury or death under various conditions [33,34]. To explore if the reduction of DCX + cells in Pb-treated animals was overtly associated with cell death, we examined the expression of active caspase-3 in the cortex by single or double labeling. Nuclear profiles were occasionally labeled in the cortex from Pb-treated or control brains using an antibody that detect in situ caspase-3 activation [35]. Double immunofluorescence failed to find clear colocalization of active caspase-3 in any DCX + cells around layer II (Figure 8D-G). In Fluoro-Jade C stain, labeled cells were occasionally encountered in the cortex in Pb-treated guinea pigs. No clear colocalization of Fluoro-Jade C and DCX was detected (Figure 8H-J). As a positive assay control, Fluoro-Jade C labeled cells were found in the rat retina 24 h following induction of acute intraocular hypotension [36], whereas no labeling existed in normal rat retina (Figure 8K-P).

## Discussion

For centuries Pb poisoning remains an environmental risk for human health in all societies [1,2,37,38], and its deleterious impact on central nervous system of children is well documented but of particular medical concern [3-11]. Children are more vulnerable to Pb exposure than adults for many potential reasons, including their exposure to Pb is favored by the habit of eating/chewing non-nutritive materials (pica habits); a child’s intestine absorbs Pb much faster than that of an adult. Another reason is that their blood–brain barrier (BBB) is not completely mature [3,6,37-39]. Blood Pb levels at or over 10-20 μg/dL in children are generally considered to cause debilitating effects on brain structure and function, manifested by neurobehavioral, learning and cognitive abnormalities [37-39]. However, recent studies have shown intellectual impairment in children with blood Pb levels below 10 μg/dL [40,41]. For adults, high Pb levels may penetrate into the brain by accumulating on the mature BBB and/or altering its functional competence [42]. In the present study, blood Pb concentrations in the experimental guinea pigs have reached over 10 μg/dL, therefore, are above the threshold levels that would cause neurotoxicity in rats and humans[12-14,39-41].

Overall, the mechanism(s) underscoring Pb-induced neurotoxicity in immature and adult brains appear to be complex, and may involve many aspects including oxidative stress, apoptosis, calcium homeostasis disruption and deregulation of cell signaling [42-49]. As one of the potential mechanistic concerns of Pb-induced neurotoxicity in the developing brain, several research groups have reported that chronic low level Pb exposure inhibit adult neurogenesis in the small laboratory rodents [12-14], which may contribute to behavioral and cognitive impairments. Consistent with these reports, we have observed a dramatic loss of DCX + neurons in both the SVZ and SGZ in young adult guinea pigs.

In general, the hippocampal formation is functionally related to behavioral and cognitive activities such as memory and learning. However, the neocortex plays an increasingly critical role in cognition in mammals during evolution in parallel with cortical expansion and the development of complicated network systems [18-23]. Several research groups (including us) have independently described the presence of DCX + cells around cortical layer II in different adult mammalian species including guinea pigs, cats, non-human primates and humans [15-26]. While the origin, fate and developmental trajectory of these cells remain to be further characterized [27-32,50], these cells can be activated by novel environmental exploration [31], which suggests that they might be involved in cortical network activity under physiological conditions. Our present results show that DCX + cells in different cortical areas in young adult guinea pigs decrease after treatment with 0.2% lead acetate for 4 and 6 months. Thus, chronic Pb exposure can result in remarkable loss of immature neurons in broad neocortical areas. Further, our present analysis also shows that loss of smaller sized DCX + cells appears more significantly relative to larger sized ones. DCX + cells with a larger somal size and more developed neuronal processes often co-localize with the neuron-specific nuclear protein (NeuN), a mature neuronal marker [17,18,24,30,32,50]. Thus, it appears that the smaller DCX + cells with a less mature phenotype may be more sensitive to Pb exposure. Because these small-sized neurons are more common than the large-sized ones in young mammals, Pb pollution could significantly impact cerebral neuronal development in children.

Hypothetically, reduction of DCX + cortical immature neurons in Pb-treated animals could be resulted from loss of cells or decline in DCX expression. The former condition could be a consequence of reduced cell genesis and/or increased cell death. It is currently controversial as to whether layer II DCX + immature are or can be generated in postnatal life due to inconsistent BrdU birth-dating data [27-32,50]. In this study we could not find a clear colocalization of BrdU in cortical DCX + neurons at 2 and 4 months after BrdU injection. The lack of active caspase-3 and Fluoro-Jade-C colocalization indicates that DCX + cortical cells in Pb-treated guinea pigs are not overtly associated with apoptotic death, nor do they appear apparently injured.

## Conclusions

This study demonstrates that Pb exposure induces a decline of DCX-expressing immature neurons in the neocortex as well as in the established neurogenic sites in adult guinea pigs, with a greater effect seen in the relatively small-sized subpopulation of the cells in the cortex. The findings from this study point to a potential new cellular substrate in mammalian cerebrum that would be expected to be vulnerable to chronic Pb exposure and toxicity in the pediatric population.

## Methods

### Animals

Thirty-six 2-month-old male guinea pigs weighing 300-350 g were used in the present study. All animals were housed in acrylic box cages and freely accessible to rodent chow and water. Animals were maintained under conditions of constant temperature (25 °C), humidity (50 ± 10%) and lighting cycle (12:12 h). Rat retinal sections from another study involving experimental glaucoma were used for assay control. All experimental procedures used in the present study were approved by the Animal Ethics Committee of Xiangya School of Medicine, Central South University, in accordance with the experimental animal use and welfare requirements set by the Ministry of Health of China as well as the NIH guidelines for use and care of laboratory animals.

### Chronic lead exposure and BrdU injection

Eighteen animals were used as Pb exposure groups, and they received 0.2% lead acetate in drinking water for 2, 4 and 6 months, respectively (n = 6/time point). The remaining 18 age-matched control animals received the same type of drinking water without the addition of Pb. In each group, half of the animals were used for morphological study, with the rest used for blood Pb level measurements. BrdU pulse-chasing study was applied to the 4 and 6 months surviving group. Thus, two BrdU injections (50 mg/kg, i.p., 8 h apart, B5002, Sigma-Aldrich, MO, USA) were given after 2 months dosing of Pb or vehicle.

### Measurement of blood Pb concentration

After surviving for 2, 4 and 6 months, animals were anesthetized with chloral hydrate (4 mg/kg, i.p.), and 100 μL of blood were collected from the left ventricle of the heart immediately following thoraxitomy. For each analysis, blood Pb concentration was measured by BH2100 graphite furnace atomic absorption spectrophotometry (Bohui, Beijing, China) spectrophotometer in the clinical laboratory of the Third Xiangya Hospital of Central South University [51]. The final concentration of blood Pb was expressed as μg/dL.

### Immunohistochemistry

Animals were anesthetized with chloral hydrate (4 mg/kg, i.p.) and then perfused with saline followed by 4% paraformaldehyde in 0.01 M phosphate-buffered saline (PBS) at 4 °C. Brains were dissected and post-fixed by immersion in the same fixative at 4 °C for 24 h, and were passed in gradual concentrations of sucrose (10%, 20% and 30%) until they sunk. The brains were cut coronally in a cryostat at 30 μm and 10 μm, with sections collected alternatively in two 12-well culture plates. For immunohistochemistry using the avidin-biotin complex method, a set of sections were treated with 1% H_2_O_2_ in 0.01 M PBS for 30 min, and pre-incubated in 5% normal horse serum (Sigma-Aldrich, MO, USA) in PBS with 0.3% Triton X-100 for 1 h at room temperature, followed by incubation with the goat anti-DCX antibody (polyclone, sc-8066, Santa-Cruz, CA, USA, 1:1000) overnight at 4 °C [15]. Sections were further reacted with a biotinylated pan-specific secondary antibody (horse anti-mouse, rabbit and goat IgG, Vector, CA, USA, 1:400) for 2 h., and subsequently with avidin-biotin complex reagents (Vector, CA, USA, 1:400) for 1 h. Immunoreaction product was visualized using 0.003% hydrogen peroxide and 0.05% diaminobenzidine. Three 10-min washes with PBS were used between incubations. Sections were mounted on microslides, allowed to air-dry, and coverslippered. The specificity of this DCX antibody was evaluated previously by multiple groups of investigators [15].

For double immunofluorescence, sections (10 μm, thaw-mounted onto slides) were blocked with 5% donkey serum (Sigma-Aldrich, MO, USA) for 30 min, and then reacted with goat anti-DCX and one of the following antibodies: rat anti-BrdU (monoclonal, MCA2060, Serotec, CA, USA, 1:2000), mouse anti-NeuN (MAB377, Millipore, MA, USA, 1:1000) and rabbit anti caspase-3 (ab3235, Abcam, MA, USA, 1:1000). Sections subjected to BrdU immunolabeling were treated in 1 X SSC and 50% formamide for 1 h at 65 °C, then in 2 N HCl for 30 min at 37 °C prior to primary antibody incubation. Immunoreaction products were visualized using Alexa Fluor 488 and 594 conjugated secondary antibodies generated in donkey (Jackson ImmunoResearch, PA, USA, 1:200). Fluoro-Jade C stain was carried out according to manufacturer’s instruction (AG325, Millipore, MA, USA). In brief, the sections were air dried at room temperature overnight after DCX reaction of immunofluorescence consummated, rehydrated for 2 min in distilled water and then transferred to the 0.06% potassium permanganate solution for 10 min. The slides were then rinsed for 2 min in distilled water, transferred to the Fluoro-Jade C working solution for 10 min and then rinsed, air dehydrated, xylene cleared and coverslipped with DPX. All sections were counterstained with DAPI (C1002, Beyotime, Beijing, China, 1:10000), washed and counterstained with DAPI before microscopic examination.

### Imaging and densitometry

Immunostained sections were examined and imaged on microscope (BX40, Olympus, Tokyo, Japan) using 4×, 10× and/or 40× objective lens. Four cortical areas were arranged for systematic imaging and densitometry, including the frontal cortex, parietal cortex, temporal cortex and occipital cortex. The methodology for cell counting with a Motic pathology picture HD analysis (Motic, Xiamen, China) was described previously [17]. In brief, DCX + cells around layer II were counted in montaged 10× images taken along the cortical surface, and cell density was calculated based on the number of labeled cells underneath unit length of the pial surface (i.e., # of cells/1 mm × 30 μm). During cell counting, DCX + cells with a longer somal diameter <10 μm (ranged from 5-10 μm) were marked with a cross sign “×”, whereas those >10 μm (ranged from 10-20 μm) marked with a small ruler bar (-) of 10 μm in length. The number of the cells was then recorded and relative density calculated after obtaining the corresponding pial length of the measured cortical area.

### Data analysis

Data were analyzed using SPSS 10.0 (SPSS, IL, USA). Means of blood Pb levels and DCX + cell densities were calculated for individual and groups of animals. Statistical comparisons were conducted using one-way ANOVA or Student’s *t*-test, with *P* ≤ 0.05 being considered statistically significance.

## Authors' contributions

KX, JFH and XXY designed the experiments. KH, MQZ, CLF and KX performed the experiments. HW and DC analyzed the data. KX, LS drafted the manuscript. XXY finalized the manuscript. All authors read and approved the final manuscript.

## References

[B1] Fuller R Lead exposures from car batteries-a global problem Environ Heal Perspect 2009 117 12 A535 10.1289/ehp.0901163 PMC2799476 20049180

[B2] Shi GT Chen ZL Xu SY Wang L Zhang J Li HW Li LN Characteristics of heavy metal pollution in soil and dust of urban parks in Shanghai Huan Jing Ke Xue 2007 28 2 238 242 17489176

[B3] Lanphear BP Childhood lead poisoning prevention: too little, too late JAMA 2005 293 18 2274 2276 15886384 10.1001/jama.293.18.2274

[B4] Brubaker CJ Dietrich KN Lanphear BP Cecil KM The influence of age of lead exposure on adult gray matter volume Neurotoxicology 2010 31 3 259 266 20226811 10.1016/j.neuro.2010.03.004 PMC2866835

[B5] Cecil KM Brubaker CJ Adler CM Dietrich KN Altaye M Egelhoff JC Wessel S Elangovan I Hornung R Jarvis K Lanphear BP Decreased brain volume in adults with childhood lead exposure PLoS Med 2008 5 5 e112 18507499 10.1371/journal.pmed.0050112 PMC2689675

[B6] Peters JL Kubzansky LD Ikeda A Spiro A Wright RO Weisskopf MG Kim D Sparrow D Nie LH Hu H Schwartz J Childhood and adult socioeconomic position, cumulative lead levels, and pessimism in later life: the VA normative aging study Am J Epidemiol 2011 174 12 1345 1353 22071587 10.1093/aje/kwr269 PMC3276297

[B7] Bellinger D Sloman J Leviton A Rabinowitz M Needleman HL Waternaux C Low-level lead exposure and children’s cognitive function in preschool years Pediatrics 1991 87 2 219 227 1987535

[B8] Bellinger D Stiles K Needleman HL Low-level lead exposure intelligence and academic achievement. A long-term follow-up study Pediatrics 1992 90 6 855 861 1437425

[B9] Bleecker ML Ford DP Lindgren KN Hoese VM Walsh KS Vaughan CG Differential effects of lead exposure on components of verbal memory Occup Environ Med 2005 62 3 181 187 15723883 10.1136/oem.2003.011346 PMC1740967

[B10] Carfield RL Gendle MH Cory-Slechta DA Impaired neuropsychological functioning in lead-exposed children Dev Neuropsychol 2004 26 1 513 540 15276907 10.1207/s15326942dn2601_8

[B11] Winneke G Kramaer U Neurobehavioral aspects of lead neurotoxicity in children Eru J Pub Health 1997 5 2 65 69 9208160

[B12] Gilbert ME Kelly ME Samsam TE Goodman JH Chronic developmental lead exposure reduced neurogenesis in adult rat hippocampus but does not impair spartial learning Toxicol Sci 2005 86 2 365 374 15788721 10.1093/toxsci/kfi156

[B13] Jaako-Movits K Zharkovsky T Romantchik O Jurgenson M Merisalu E Heidmets LT Zharkovsky A Developmental lead exposure impairs contextual fear conditioning and reduces and reduces and hippocampus neurogenesis in the rat brain Int J Dev Neurosci 2005 23 7 627 635 16150564 10.1016/j.ijdevneu.2005.07.005

[B14] Verina T Rohde CA Guilarte TR Environmental lead exposure during early life alters granule cell neurogenesis and morphology in the hippocampus of young adult rats Neuroscience 2007 145 3 1037 1047 17276012 10.1016/j.neuroscience.2006.12.040 PMC1892316

[B15] Nacher J Crespo C McEwen BS Doublecortin expression in the adult rat telencephalon Eur J Neurosci 2001 14 4 629 644 11556888 10.1046/j.0953-816x.2001.01683.x

[B16] Liu YW Curtis MA Gibbons HM Mee EW Bergin PS Teoh HH Connor B Dragunow M Faull RL Doublecortin expression in the normal and epileptic adult human brain Eur J Neurosci 2008 28 11 2254 2265 19046368 10.1111/j.1460-9568.2008.06518.x

[B17] Xiong K Luo DW Patrylo PR Luo XG Struble RG Clough RW Yan XX Doublecortin-expressing cells are present in layer II across the adult guinea pig cerebral cortex: partial colocalization with mature interneuron markers Exp Neurol 2008 211 1 271 282 18378231 10.1016/j.expneurol.2008.02.003 PMC2994188

[B18] Cai Y Xiong K Chu Y Luo DW Luo XG Yuan XY Struble RG Clough RW Spencer DD Williamson A Kordower JH Patrylo PR Yan XX Doublecortin expression in adult cat and primate cerebral cortex relates to immature neurons that develop into GABAergic subgroups Exp Neurol 2009 216 2 342 356 19166833 10.1016/j.expneurol.2008.12.008 PMC2902881

[B19] Zhang XM Cai Y Chu Y Chen EY Feng JC Luo XG Xiong K Struble RG Clough RW Patrylo PR Kordower JH Yan XX Doublecortin-expressing cells persist in the associative cerebral cortex and amygdala in aged nonhuman primates Front Neuroanat 2009 3 17 19862344 10.3389/neuro.05.017.2009 PMC2766270

[B20] Luzzati F Bonfanti L Fasolo A Peretto P DCX and PSA-NCAM expression identifies a population of neurons preferentially distributed in associative areas of different pallial derivatives and vertebrate species Cereb Cortex 2009 19 5 1028 1041 18832334 10.1093/cercor/bhn145

[B21] Srikandarajah N Martinian L Sisodiya SM Squier W Blumcke I Aronica E Thom M Doublecortin expression in focal cortical dysplasia in epilepsy Epilepsia 2009 50 12 2619 2628 19583780 10.1111/j.1528-1167.2009.02194.x

[B22] Bloch J Kaeser M Sadeghi Y Rouiller EM Redmond DE Brunet JF Doublecortin-positive cells in the adult primate cerebral cortex and possible role in brain plasticity and development J Comp Neurol 2011 519 4 775 789 21246554 10.1002/cne.22547

[B23] Marlatt MW Philippens I Manders E Czéh B Joels M Krugers H Lucassen PJ Distinct structural plasticity in the hippocampus and amygdala of the middle-aged common marmoset (Callithrix jacchus) Exp Neurol 2011 230 2 291 301 21605555 10.1016/j.expneurol.2011.05.008

[B24] Zhang J Giesert F Kloos K Vogt Weisenhorn DM Aigner L Wurst W Couillard-Despres S A powerful transgenic tool for fate mapping and functional analysis of newly generated neurons BMC Neurosci 2011 11 158 21194452 10.1186/1471-2202-11-158 PMC3019205

[B25] Fung SJ Joshi D Allen KM Sivagnanasundaram S Rothmond DA Saunders R Noble PL Webster MJ Weickert CS Developmental patterns of doublecortin expression and white matter neuron density in the postnatal primate prefrontal cortex and schizophrenia PLoS One 2011 6 9 e25194 21966452 10.1371/journal.pone.0025194 PMC3180379

[B26] Haynes RL Xu G Folkerth RD Trachtenberg FL Volpe JJ Kinney HC Potential neuronal repair in cerebral white matter injury in the human neonate Pediatr Res 2011 69 1 62 67 20924315 10.1203/PDR.0b013e3181ff3792 PMC3282988

[B27] Bernier PJ Bedard A Vinet J Levesque M Parent A Newly generated neurons in the amygdala and adjoining cortex of adult primates Proc Natl Acad Sci USA 2002 99 17 11464 11469 12177450 10.1073/pnas.172403999 PMC123279

[B28] Pekcec A Löscher W Potschka H Neurogenesis in the adult rat piriform cortex Neuroreport 2006 17 6 571 574 16603913 10.1097/00001756-200604240-00003

[B29] Shapiro LA Ng KL Kinyamu R Whitaker-Azmitia P Geisert EE Blurton-Jones M Zhou QY Ribak CE Origin, migration and fate of newly generated neurons in the adult rodent piriform cortex Brain Struct Funct 2007 212 2 133 148 17764016 10.1007/s00429-007-0151-3

[B30] Guo F Maeda Y Ma J Xu J Horiuchi M Miers L Vaccarino F Pleasure D Pyramidal neurons are generated from oligodendroglial progenitor cells in adult piriform cortex J Neurosci 2010 30 36 12036 12049 20826667 10.1523/JNEUROSCI.1360-10.2010 PMC2940828

[B31] Xiong K Cai Y Zhang XM Huang JF Liu ZY Fu GM Feng JC Clough RW Patrylo PR Luo XG Hu CH Yan XX Layer I as a putative neurogenic niche in young adult guinea pig cerebrum Mol Cell Neurosci 2010 45 2 180 191 20599617 10.1016/j.mcn.2010.06.009 PMC2923265

[B32] Varea E Belles M Vidueira S Blasco-Ibáñez JM Crespo C Pastor AM Nacher J PSA-NCAM is expressed in immature, but not recently generated, neurons in the adult cat cerebral cortex layer II Front Neurosci 2011 5 17 21415912 10.3389/fnins.2011.00017 PMC3042688

[B33] Lamkanfi M Festjens N Declercq W Vanden Berghe T Vandenabeele P Caspases in cell survival, proliferation and differentiation Cell Death Differ 2007 14 1 44 55 17053807 10.1038/sj.cdd.4402047

[B34] Kutsuna N Suma T Takada Y Yamashita A Oshima H Sakatani K Yamamoto T Katayama Y Decrease in doublecortin expression without neuronal cell death in rat retrosplenial cortex after stress exposure Neuroreport 2012 23 4 211 215 22198688 10.1097/WNR.0b013e32834fca3a

[B35] Yan XX Najbauer J Woo CC Dashtipour K Ribak CE Leon M Expression of active caspase-3 in mitotic and postmitotic cells of the rat forebrain J Comp Neurol 2001 433 1 4 22 11283945 10.1002/cne.1121

[B36] Chen D Tong JB Wang H Zeng LP Zhou J Huang JF Luo XG Synaptophysin expression in rat retina following acute high intraocular pressure Acta Histochem Cytochem 2008 41 6 173 178 19180202 10.1267/ahc.08034 PMC2629489

[B37] Needleman HL Low level lead exposure: a continuing problem Pediatr Ann 1990 19 13 208 214 2181392 10.3928/0090-4481-19900301-08

[B38] Taylor MP Schniering CA Lanphear BP Jones AL Lessons learned on lead poisoning in children: one-hundred years on from Turner's declaration J Paediatr Child Health 2010 47 849 856 20598069 10.1111/j.1440-1754.2010.01777.x

[B39] Needleman HL Gatsonic CA Low-level lead exposure and the IQ of children. A meta-analysis of modern studies JAMA 1990 263 5 673 878 2136923

[B40] Canfield RL Henderson CR Cory-Slechta DA Cox C Jusko TA Lanphear BP Intellectual impairment in children with blood lead concentration below 10 microg per deciliter N Engl J Med 2003 348 16 1517 1526 12700371 10.1056/NEJMoa022848 PMC4046839

[B41] Chiodo LM Covington C Sokol RJ Hannigan JH Jannise J Ager J Greenwald M Delaney-Black V Blood lead levels and specific attention effects in young children Neurotoxicol Teratol 2007 29 5 538 546 17553667 10.1016/j.ntt.2007.04.001

[B42] Goldstein GW Brain capillaries: a target for inorganic lead poisoning Neurotoxicology 1984 5 3 167 175 6542976

[B43] Goldstein GW Evidence that lead acts are as a calcium substitute in second messenger metabolism Neurotoxicology 1993 14 2–3 97 101 8247416

[B44] Fox DA He L Poblenz AT Medrano CJ Blocker YS Srivastava DL Lead-induced alterations in retinal cGMP phosphodiesterase trigger calcium overload, mitochondrial dysfunction and rod photoreceptor apoptosis Toxicol Lett 1998 102-103 359 361 10022279 10.1016/s0378-4274(98)00232-x

[B45] Flora SJ Saxena G Mehta A Reversal of lead-induced neuronal apoptosis by chelation treatment in rats: role of reactive oxygen species and intracellular Ca (2+) J Pharmacol Exp Ther 2007 322 1 108 116 17431133 10.1124/jpet.107.121996

[B46] Sharifi AM Mousavi SH Jorjani M Effect of chronic lead exposure on pro-apoptotic Bax and anti-apoptotic Bcl-2 protein expression in rat hippocampus in vivo Cell Mol Neurobiol 2010 30 5 769 774 20148304 10.1007/s10571-010-9504-1 PMC11498842

[B47] Nava-Ruíz C Alcaraz-Zubeldia M Méndez-Armenta M Vergara P Díaz-Ruìz A Ríos C Nitric oxide synthase immunolocalization and expression in the rat hippocampus after sub-acute lead acetate exposure in rats Exp Toxic Pathology 2010 62 3 311 316 10.1016/j.etp.2009.04.006 19524414

[B48] Tavakoli-Nezhad M Barron AJ Pitts DK Postnatal inorganic lead exposure decreases the number of spontaneously active midbrain dopamine neurons in the rat Neurotoxicology 2001 22 2 259 269 11405257 10.1016/s0161-813x(01)00010-9

[B49] Oberto A Marks N Evans HL Guidotti A Lead promotes apoptosis in newborn rat cerebellar neurons: pathological implications J Pharmacol Exp Ther 1996 279 1 435 442 8859023 10.1163/2211730x96x00234

[B50] Klempin F Kronenberg G Cheung G Kettenmann H Kempermann G Properties of doublecortin-(DCX)-expressing cells in the piriform cortex compared to the neurogenic dentate gyrus of adult mice PLoS One 2011 6 10 e25760 22022443 10.1371/journal.pone.0025760 PMC3192736

[B51] Huang JF Huang K Shang L Wang H Yan XX Xiong K Beta-amyloid precursor protein cleavage enzyme-1 expression in adult rat retinal neurons in the early period after lead exposure Neural Regen Res 2011 6 1045 1051

